# How Effective Are Noninvasive Tests for Diagnosing Malignant Peripheral Nerve Sheath Tumors in Patients with Neurofibromatosis Type 1? Diagnosing MPNST in NF1 Patients

**DOI:** 10.1155/2019/4627521

**Published:** 2019-07-01

**Authors:** Maria Schwabe, Stanislav Spiridonov, Elizabeth L. Yanik, Jack W. Jennings, Travis Hillen, Maria Ponisio, Douglas J. McDonald, Farrokh Dehdashti, Cara A. Cipriano

**Affiliations:** Washington University School of Medicine in St. Louis, 660 South Euclid Avenue, St. Louis, MO 63110, USA

## Abstract

**Background:**

Distinguishing between benign and malignant peripheral nerve sheath tumors (MPNSTs) in neurofibromatosis 1 (NF1) patients prior to excision can be challenging. How can MPNST be most accurately diagnosed using clinical symptoms, magnetic resonance imaging (MRI) findings (tumor size, depth, and necrosis), positron emission tomography (PET) measures (SUV_peak_, SUV_max_, SUV_max tumor_/SUV_mean liver_, and qualitative scale), and combinations of the above? *Methods*. All NF1 patients who underwent PET imaging at our institution (January 1, 2007–December 31, 2016) were included. Medical records were reviewed for clinical findings; MR images and PET images were interpreted by two fellowship-trained musculoskeletal and nuclear medicine radiologists, respectively. Receiver operating characteristic (ROC) curves were created for each PET measurement; the area under the curve (AUC) and thresholds for diagnosing malignancy were calculated. Logistic regression determined significant predictors of malignancy.

**Results:**

Our population of 41 patients contained 34 benign and 36 malignant tumors. Clinical findings did not reliably predict MPNST. Tumor depth below fascia was highly sensitive; larger tumors were more likely to be malignant but without a useful cutoff for diagnosis. Necrosis on MRI was highly accurate and was the only significant variable in the regression model. PET measures were highly accurate, with AUCs comparable and cutoff points consistent with prior studies. A diagnostic algorithm was created using MRI and PET findings.

**Conclusions:**

MRI and PET were more effective at diagnosing MPNST than clinical features. We created an algorithm for preoperative evaluation of peripheral nerve sheath tumors in NF1 patients, for which additional validation will be indicated.

## 1. Introduction

Neurofibromatosis type 1 (NF1) is one of the most common autosomal-dominant diseases worldwide [1–8]. It has an incidence of 1/2,500 to 1/3,500 individuals [1–5, 8, 9] and is caused by mutations of the NF1 gene located on chromosome 17q11.2. Clinically, the disease is characterized by multiple plexiform neurofibromas that are usually benign; however, they have the potential for malignant transformation, with a lifetime risk of 8–12% [1–5, 8]. Malignant peripheral nerve sheath tumors (MPNSTs) are the leading cause of mortality in NF1, reducing average life expectancy by 10–15 years [2, 6, 10, 11].

Differentiating between benign and malignant PNSTs can be challenging, especially in individuals with multiple neurofibromas. Traditionally, this has been attempted based on imaging characteristics and symptoms, which may include pain, increasing size of a mass, and new neurological deficit [5]. However, there is significant overlap in the appearance as well as clinical manifestations of benign and malignant tumors [5, 12].

**Figure 1:**
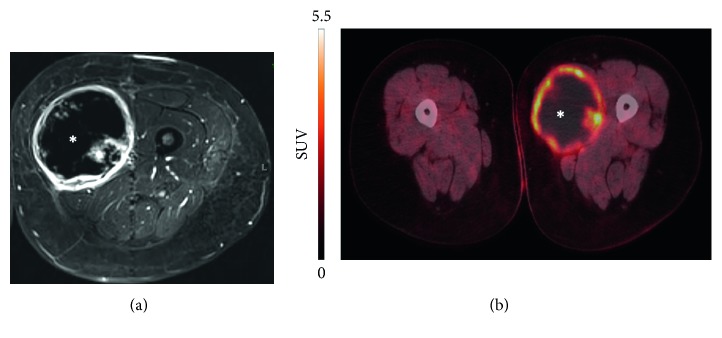
Example of a malignant peripheral nerve sheath tumor in the adductor musculature of the left thigh visualized using (a) magnetic resonance imaging (MRI) and (b) positron emission tomography (PET) studies. The hypointense areas marked with the asterisk represent necrosis within the tumor.

Several magnetic resonance imaging (MRI) features can be useful in distinguishing MPNSTs from neurofibromas. These include largest dimension of the mass, heterogeneity indicating necrosis, peripheral enhancement pattern, perilesional edema-like zone, intratumoral cystic lesion, and irregular margins [12, 13]. Of these features, tumor size and necrosis are the best supported predictors for diagnosing MPNST [12, 14–17] (Figure 1(a)).

Multiple efforts have been made to accurately diagnose malignant transformation using metabolic imaging with positron emission tomography/computed tomography (PET/CT) and [^18^F]fluorodeoxyglucose (FDG) (Figure 1(b)). Several authors have studied both semiquantitative and qualitative methods of evaluating lesions with overall good success [8, 18, 19]. Parameters for semiquantitative analysis include but are not limited to mean standardized uptake value (SUV_mean_), maximum SUV (SUV_max_), maximum SUV corrected for lean body mass (SUL_max_), and various ratios comparing tumor FDG avidity to that of other tissues, such as the liver, muscle, and fat. Among the most common of these ratios is SUV_max tumor_/SUV_mean liver_, also referred to as the tumor-to-liver ratio (TLR). Semiquantitative methods for diagnosing MPNST, such as mean SUV_max_, have sensitivities of 94%–100% and specificities of 76%–94% [18]. Similarly, qualitative methods, such as visual descriptions of hypermetabolic lesions, have yielded sensitivities of 91%–100% and specificities of 67%–95% [18, 20, 21].

In spite of this research, to our knowledge, a noninvasive gold standard algorithm for diagnosing MPNST has not been established, nor have imaging modalities been evaluated in combination with clinical features. The aim of our retrospective study was to develop a strategy for distinguishing benign from malignant PNST using noninvasive observations and tests, specifically clinical symptoms, MRI features (size, depth, and necrosis), PET measures (SUV_peak_, SUV_max_, SUV_max tumor_/SUV_mean liver_, and qualitative scale), and combinations of the above.

## 2. Materials and Methods

### 2.1. Patient Population and Data Collection

Following IRB approval, all patients with a diagnosis of NF1 who were treated at our institution between 1 January 2007 and 31 December 2016 were identified by searching the medical oncology, nuclear medicine, and surgical databases at our institution. Our study included all patients who underwent FDG-PET/CT to evaluate for potential malignant transformation of a PNST with available imaging and confirmed histopathology. Electronic medical records were reviewed for demographic information (patient age at time of surgery, gender, and tumor location) as well as potential predictors for malignancy. These included preoperative MRI features (tumor size, tumor depth relative to the fascia, and necrosis) [22], PET imaging measures (SUV_max_, SUV_peak_, and SUV_max tumor_/SUV_mean liver_), and clinical findings (pain, enlargement, and nerve symptoms). Histopathology results from biopsy or surgery obtained through chart review were used as the gold standard for diagnosing benign versus malignant PNST.

### 2.2. MRI Analysis

MR images were assessed by two fellowship-trained musculoskeletal radiologists blinded to diagnoses. Tumor size was measured as the largest diameter on T1 sequences and analyzed as both a continuous and categorical variable, the latter using 5 cm as a cutoff based on the AJCC staging system. Tumor depth was analyzed as a categorical variable, either superficial or deep to the fascia. Necrosis was defined as nonenhancement on T1 fat-saturated postcontrast images, often with increased T2 signal intensity, and recorded in quartiles (0%, <25%, 25–49%, 50–74%, and ≥75% necrosis).

### 2.3. FDG-PET/CT Protocol

Patients were imaged according to our institution's standard protocol for FDG-PET/CT, which has previously been described [22]. In brief, patients fasted for at least 4 hours prior to FDG injection. Blood glucose levels were required to be ≤200 mg/dL prior to FDG administration. Patients were injected with approximately 10–15 mCi (370–555 MBq) of FDG. The PET/CT acquisition was started approximately 60 minutes after FDG injection. Patients were scanned from the base of the skull to the upper-thigh with extension to extremities based on the location of the lesion of the interest. Noncontrast CT images were obtained first for attenuation correction and for fusion with the PET images for lesion localization. PET images were acquired at typically 6–8 bed positions, with an acquisition time of 2–5 minutes per bed position.

### 2.4. Semiquantitative Analysis

SUVs were measured using the commercial software Hermes (Hermes Medical Solutions, Sweden) by placing a volume of interest (VOI) with a diameter of 1.5 cm over the most intense region of the lesion on the axial PET/CT images. For SUV_max_, the highest SUV and for SUV_peak_, the average of the highest SUV within the VOIs were measured and recorded. When necessary, the diameter of the sphere was adjusted to accommodate for lesion size. Mean liver SUV (average SUV within the VOIs) was measured using the same 1.5 cm diameter sphere placed over the right lobe of the liver. TLR was calculated using SUV_max_ of the tumor over SUV_mean_ of the liver.

### 2.5. Qualitative Analysis

FDG-PET/CT images were evaluated by one nuclear medicine physician and one nuclear medicine fellow who had completed a diagnostic radiology residency. Sites of abnormal metabolic activity were scored on a 5-point scale based on the following criteria: score of 1 = uptake similar to background, score of 2 = uptake greater than background but less than mediastinal blood pool (MBP), score of 3 = uptake > MBP but less than or equal to liver, score of 4 uptake > liver, and score of 5 = uptake markedly > than liver (greater than 2-3 times).

### 2.6. Statistical Analysis

Tumor necrosis was analyzed as a categorical variable, once with any necrosis considered a positive finding and once with necrosis >25% considered a positive finding. For each PET measurement, receiver operating characteristic curves (ROC) were created, and the area under the curve (AUC) was calculated, along with cutoff points for diagnosing malignancy that optimized sensitivity and specificity. When these were conflicting, sensitivity was prioritized in order to minimize false negative results in the context of evaluating for malignancy. These cutoff points and their sensitivity/specificity in our dataset were compared to cutoff points previously reported in the literature. The sensitivity, specificity, positive predictive value (PPV), and negative predictive value (NPV) were calculated for each tumor characteristic, imaging measure, and clinical finding, and relevant combinations of these variables. Logistic regression was used to evaluate variables for predicting malignancy in combination. Superficial tumors were not included in the model because no malignancies were observed within this group. Potential predictors (tumor size, necrosis, PET parameters, and clinical findings) were selected for entry into the model based on the results of the accuracy analysis above.

### 2.7. Diagnostic Algorithm

Our aggregate results were used to create an algorithm for suggested evaluation of PNST in NF1 patients. In order to develop a clinically relevant workup strategy, tumors were first differentiated by tumor features that can be determined by history and physical examination, then by noninvasive imaging studies, and finally by biopsy. For each branch point, we calculated the NPV and PPV to inform clinicians about the likelihood of malignancy given the available information. Lastly, we used the PPV from the noninvasive workup to offer recommendations for management following biopsy, taking pretest probability into account.

## 3. Results

Our population of 41 patients contained 34 benign and 36 malignant tumors. The mean patient age was 30 years old for the overall population (range 9–62). Within the 41 patients, there was a predominance of tumors in females (41) compared to males (29). The majority of tumors were deep and axially located, and the mean diameter was 6.5 cm (range 1.5–20.0 cm). Mean SUV_max_, SUV_peak_, and TLR were 7.9 (standard deviation (SD) ± 5.4), 6.4 (SD ± 4.3), and 4.0 (SD ± 2.6), respectively. Necrosis was evident on MRI in 19 cases (51.4%). Most patients reported pain and enlargement of tumors but not associated nerve symptoms. Additional descriptive statistics including demographic information and potential predictors of malignancy are summarized in Table 1.

### 3.1. Clinical Findings

Clinical findings were available in 41 patients for 60 of the 70 tumors. Among patient symptoms, we found that pain and enlargement were more sensitive (sensitivity 85.2% and 85.2%; specificity 28.1% and 25.0%, respectively), while nerve symptoms were more specific (sensitivity 44.8%; specificity 75.0%). Combining nerve symptoms and either pain or growth did not improve diagnostic accuracy compared to evaluation of nerve symptoms alone. Of note, having any two of three symptoms was more specific and slightly more sensitive than having any one particular symptom. Having all three symptoms was insensitive and only moderately specific and therefore not diagnostically useful (Table 2).

### 3.2. MRI Findings

Fifty-nine lesions had PET/CT images, of which 37 had MRI images available for review. Tumor depth below the fascia was highly sensitive for malignancy (100%) with 100% NPV, but not specific (20.7%). Increasing tumor size was predictive of malignancy as a continuous variable in regression analysis; however, the cutoff of 5 cm (based on the AJCC system) was neither sensitive nor specific. An ROC curve was also constructed for tumor size, but with AUC 0.687 (CI 0.539–0.835), there was no clear threshold for diagnosing malignancy.

Between the two musculoskeletal radiologists reviewing MRI studies, interobserver agreement was good (kappa 0.608, 95% CI 0.409–0.807) for necrosis by quartiles (none, 0–25%, 25–50%, etc.). Agreement was very good for no necrosis versus any necrosis (kappa 0.937, 95% CI: 0.816–1.000) and necrosis less than versus greater than 25% (kappa 0.852, 95% CI: 0.655–1.000). For diagnosing MPNST, any necrosis seen on MRI was sensitive (87.5%) and fairly specific (76.1%), whereas necrosis of >25% of the tumor volume was less sensitive (75.0%) but more specific (95.2%), with PPV 92.3%.

### 3.3. PET Findings

The ROC curves for SUV_max_, SUV_peak_, and TLR were noted to have similar AUCs (Table 3). Optimal cutoff points were chosen to maximize sensitivity and specificity based on our patient population. Our cutoff points for SUV_peak_ (4.5) and TLR (3.0) were identical to those in the prior literature, and our cutoff for SUV_max_ was similar (5.3 in our data compared to 5.0 in the literature, with some more conservative cutoffs slightly lower) [13, 23, 24]. In our dataset, the previously established cutoff of 5.0 resulted in identical sensitivity but lower specificity (60% compared to 70%) than our optimal cutoff point of 5.3 (Table 3).

For qualitative assessment, the interobserver agreement was very good, with kappa 0.896, 95% CI 0.740–1.000. Level 5/5 on the visual scale was considered suggestive of malignancy. Using these thresholds for diagnosing MPNST, PET measures were comparable to one another, with generally good predictive value. Of these, SUV_peak_ > 4.5 had the highest sensitivity and NPV and was therefore selected for testing in combination with MRI necrosis. Positive findings on either MRI (any necrosis) or PET (SUV_peak_ > 4.5) were highly sensitive (95.5%), while positive findings on both MRI and PET were highly specific (93.9%).

Logistic regression analysis supported the value of necrosis and PET measures for diagnosing MPNST. Depth was found to have 100% sensitivity, with all malignancies occurring deep to the fascia in our population; therefore, only 27 deep tumors were included for the development of prediction models. In order to prevent multicollinearity between similar measures, SUV_peak_ > 4.5 was selected to represent PET measures, and necrosis >25% of the tumor volume was chosen to represent MRI findings. Thus, tumor size, necrosis >25%, and SUV_peak_ were selected as the relevant variables; when these were entered for deep tumors, necrosis was the only significant predictor (*p*=0.033, Nagelkerke *R*^2^ = 0.622).

### 3.4. Combined Diagnostic Algorithm

Clinical findings were not included in the algorithm as they could not reliably rule out malignancy in our population (NPV 62.3–69.2%). SUV_peak_ was included because it had the highest sensitivity and NPV of the semiquantitative PET parameters; however, the visual scale was almost identical, and the other PET measures were not significantly different, so these could be substituted for SUV_peak_ with minimal effect.

**Figure 2:**
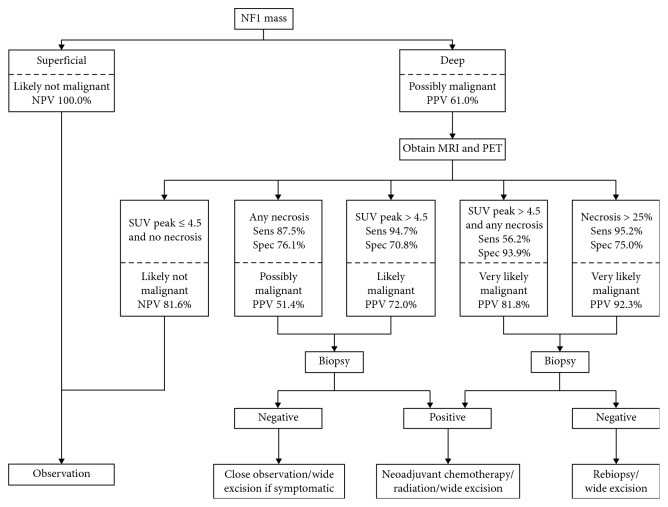
Suggested algorithm for the evaluation and management of PNST concerning for malignancy in patients with NF1. It should be noted that all predictive values are based solely on our patient population (for confidence intervals, please refer to Table 2) and the algorithm therefore requires further validation.

Tumors were first differentiated by depth relative to the fascia, which was notable for 100% NPV in our analysis. While tumor depth may be verified on MRI (as in our methods), in most cases, it can be easily determined by physical examination prior to advanced imaging. We therefore suggest that patients appearing to have deep tumors on physical examination be evaluated with MRI and PET and that they undergo biopsy in the presence of concerning features on either of these studies. Tumors that are histologically confirmed to be malignant should be managed with surgery and neoadjuvant/adjuvant chemotherapy/radiation according to standard protocols. Unfortunately, biopsy itself is imperfect due to sampling error [25]. For tumors that are likely malignant based on imaging but do not contain evidence of MPNST on initial biopsy, rebiopsy or wide excision may be indicated; in contrast, observation may be acceptable for tumors that appear less concerning (Figure 2). Of note, this algorithm is based solely on our patient population and will therefore require further validation.

## 4. Discussion

The aim of our study was to report the diagnostic value of tumor size and depth, MRI features, PET measures, and clinical findings to distinguish between benign and malignant PNST. In sum, PET measures and necrosis on MRI were the most predictive of malignancy and can be combined to direct workup and treatment in this challenging clinical situation. This algorithm has been adopted at our institution and will require validation with long-term follow-up from multiple centers.

Our study had several limitations. It is a retrospective analysis with a relatively small sample size due to the rarity of NF1 and MPNST. Most importantly, PET/CT, MRI, and clinical findings were not available for all patients; however, these missing data did not correlate with year, age, or malignancy characteristics. In addition, PET/CT studies were performed on different scanners at our institution, potentially resulting in a small amount of measurement variability that was likely not clinically relevant. Lastly, our algorithm was developed based on a limited patient population at a single institution, so external validation will be needed.

### 4.1. Clinical Findings

Traditional teaching states that most plexiform neurofibromas are asymptomatic, unless traumatized or compressed, while MPNSTs are usually associated with significant pain [8], but we are unaware of any evidence supporting this. In our patient population, pain and growth were more sensitive and nerve symptoms were more specific for diagnosing MPNST. However, either in isolation or combination with one another, clinical findings were not as predictive as imaging.

### 4.2. MRI Findings

In the AJCC staging system, sarcomas greater than 5 cm in diameter are considered at higher risk for local progression and metastasis. This is generally accepted and well supported in the MPNST literature. Of note, the most recent AJCC Cancer Staging Manual removed depth of tumor notation from the guidelines; however, assessment of tumor depth still remains prominent in the literature [26]. This is generally accepted and well supported in the MPNST literature. Kar et al. [27] found that 92% of malignant tumors were >5 cm and deep to the fascia, while Hwang et al. [28] reported that 57% of malignant tumors were >5 cm and 88% were deep to the fascia. We found that increasing tumor size was predictive of malignancy as a continuous variable in the regression analysis, but the 5 cm AJCC cutoff was neither sensitive nor specific. In addition, ROC analysis did not identify a clear size above which tumors were more likely to be malignant, which limits the clinical utility of this variable in diagnosing MPNST. All of our malignant tumors were deep to the fascia, resulting in sensitivity and NPV of approximately 100%. Our patient population did not include any superficial MPNSTs. Consistent with this, prior literature suggests that cutaneous neurofibromas do not have malignant potential, and subcutaneous neurofibromas are often symptomatic but very rarely malignant [11, 29]. Therefore, our recommendation would be to observe subcutaneous neurofibromas and consider excision if they are growing or symptomatic. In our data, tumor depth had poor specificity for malignancy (21%), consistent with prior studies [30].

Necrosis, which often results from rapid tumor growth, generally indicates aggressive behavior in sarcomas. The French or FNCLCC system utilizes histologic necrosis, along with differentiation and mitoses, to define tumor grade [31, 32]. MRI findings of necrosis have also been associated with MPNST [12]. Consistent with this, necrosis visualized on MRI was highly predictive of malignancy in our population. Necrosis greater than 25% was the most specific test for malignancy in our study (specificity 95.2%; sensitivity 75.0%), while any necrosis was less specific but sensitive (specificity 76.1%; sensitivity 87.5%). In addition, necrosis was significant in several iterations of the regression model. Thus, necrosis on MRI may be an indication for biopsy; furthermore, if histologic necrosis is noted in an otherwise nondiagnostic tissue specimen, rebiopsy or wide excision of the tumor should be strongly considered.

### 4.3. PET Findings

Several semiquantitative measures of PET images have been evaluated and compared for the diagnosis of MPNST. A review by Treglia et al. found FDG-PET/CT to be a highly sensitive noninvasive method to identify malignant change in NF1 tumors [24]. SUV_max_ has been widely used, and most studies report thresholds of <2.5 for benign and >3.5 for malignant lesions; however, the range of 2.5–3.5 remains indeterminate [24, 33]. Salamon et al. [34] found SUV_max_ threshold of >3.5 was sensitive but produced a relatively high rate of false positives, while TLR was more specific with a threshold > 2.6. SUV_peak_ has also been studied, with benign tumors ranging from 0.72–3.04 and malignant tumors from 2.41–23.38 [23]. Our analysis resulted in optimal cutoff values that were comparable to those in the prior literature for these parameters (Table 3). In contrast to other studies, we found similar predictive properties among the quantitative PET measures, with no advantage of TLR over SUV_peak_ and SUV_max_ (Table 2). Furthermore, a recent study combined PET/MRI and found similar results, with the benefits of less radiation and superior imaging than combined PET/CT. This may be another possible modality for the future [35].

Qualitative interpretation of PET imaging has also been studied in the context of PNST. In a study by Fischer et al., lesions with increased uptake were identified and rated on a five-point visual scale. They concluded that PET imaging can predict growth of PNST but did not examine the relationship between growth and malignancy [19]. Chirindel et al. also performed a qualitative analysis of PET studies in which lesions were visually assessed and dichotomized as either suspected malignant or benign. On early images (performed at 1 hour after FDG administration) the sensitivity, specificity, PPV, and NPV were 91%, 84%, 67%, and 96%, respectively, which are comparable to our findings (94%, 71%, 70%, and 94%, respectively) [18]. These results suggest that qualitative PET measures may be as accurate as semiquantitative measures in diagnosing MPNST.

## 5. Conclusion

Our results confirmed that larger tumors are more likely to be malignant, although we found no clinically relevant size threshold, and that MPNSTs superficial to the fascia are extremely rare. Metabolic measurements were relatively accurate and comparable to one another diagnostically. Clinical symptoms may also be helpful, although the limitations of their predictive properties should be understood and taken into consideration. Finally, necrosis may be a valuable and thus far underutilized predictor of malignancy.

## Figures and Tables

**Table 1:** Descriptive statistics and potential predictors of malignancy for the 70 peripheral nerve sheath tumors in our study population of 41 NF1 patients. These included patient characteristics (age and gender), PET findings (SUV_max_, SUV_peak_, and TLR), MRI findings (size, depth relative to the fascia, and necrosis) and clinical findings (pain, tumor enlargement, and nerve symptoms), and histologic diagnosis.

Patient characteristics	Age (years)	Mean	30
		Median	28
		Range	9–62
	Gender (# of tumors each)	Female	41
		Male	29

Clinical findings	Pain	Yes	46
		No	14
	Enlargement	Yes	46
		No	14
	Nerve symptoms	Yes	23
		No	37

MRI findings	Size (cm)	Mean	6.5
		Median	5.4
		Range	1.5–20.0
	Depth (relative to fascia)	Superficial	7
		Deep	63
	Necrosis	Yes	19
		No	18

PET findings	SUV_max_	Mean	7.9
		Median	7.4
		Range	0.7–22.6
	SUV_peak_	Mean	6.4
		Median	5.0
		Range	0.5–18.7
	TLR	Mean	4.0
		Median	3.7
		Range	0.4–11.9

Histology	Malignant	Yes	36
		No	34

**Table 2:** Sensitivity, specificity, positive predictive values, and negative predictive values, along with their associated confidence intervals, for potential predictors of malignancy. These included tumor characteristics (depth relative to the fascia, and size > 5 cm in diameter), MRI findings (any necrosis, or necrosis >25% by volume), PET findings (SUV_max_ > 5, SUV peak > 4.5, and TLR > 3), and clinical findings (pain, enlarging, and nerve symptoms). Combinations of imaging findings (SUV_peak_ > or < 4.5 on PET and any or >25% necrosis on MRI) and clinical findings (1 of 3, 2 of 3, or 3 of 3 symptoms) are also included.

		Sensitivity	Specificity	PPV	NPV	Accuracy
Tumor characteristics	Deep	100.0 (88.0–100.0)	20.7 (8.7–40.3)	61.0 (47.4–73.2)	100.0 (51.7–100.0)	64.6
	Size > 5 cm	68.0 (46.4–84.2)	53.5 (34.2–72.0)	56.7 (37.7–74.0)	65.2 (42.8–82.8)	60.4

MRI findings	Necrosis (any)	87.5 (60.4–97.8)	76.1 (52.4–90.9)	51.4 (34.7–67.8)	73.7 (32.2–65.3)	81.1
	Necrosis (>25%)	75.0 (47.4–91.7)	95.2 (74.1–99.8)	92.3 (62.1–100)	83.3 (61.8–94.5)	86.5

PET findings	SUV_max_ > 5	89.3 (70.6–97.2)	73.3 (50.8–87.0)	75.8 (57.4–88.2)	88.0 (67.7–96.8)	78.0
	SUV_peak_ > 4.5	94.7 (71.9–99.7)	70.8 (48.8–86.6)	72.0 (50.4–87.1)	94.4 (70.6–99.7)	78.0
	TLR > 3	91.7 (71.5–98.5)	73.9 (51.3–88.90	78.6 (58.5–91.0)	89.5 (65.5–98.2)	82.1
	Visual score	94.1 (69.2–99.7)	70.8 (48.7–86.6)	69.6 (47.0–86.0)	94.4 (70.6–99.7)	80.5

Combined PET/MRI findings	SUV_peak_ > 4.5 (PET) or any necrosis (MRI)	95.5 (75.1–100)	66.7 (47.1–82.1)	67.7 (48.5–82.7)	95.2 (74.1–100)	78.8
	SUV_peak_ > 4.5 (PET) and any necrosis (MRI)	56.2 (30.6–79.2)	93.9 (78.4–98.9)	81.8 (47.8–96.8)	81.6 (65.1–91.7)	81.6
	SUVpeak > 4.5 (PET) or necrosis >25% (MRI)	72.2 (46.5–90.3)	50.0 (26.0–74.0)	60.1 (36.7–78.5)	64 (42.8–81.2)	61.1
	SUVpeak > 4.5 (PET) and necrosis >25% (MRI)	61.5 (31.6–86.14)	100 (81.5–100)	100 (59.8–100)	78.3 (64.4–87.6)	83.9

Clinical findings	Pain	85.2 (65.4–95.1)	28.1 (14.4–47.0)	50 (35.1–64.9)	69.2 (38.9–89.6)	54.2
	Growth	85.2 (65.4–95.1)	25.0 (12.1–43.8)	48.9 (34.3–63.7)	66.7 (35.4–88.7)	52.5
	Nerve symptoms	44.8 (26.9–64.0)	75.0 (57.5–87.3)	59.1 (36.7–78.5)	62.3 (46.7–76.6)	61.0

Combined clinical findings	1 of 3 findings	92.9 (75.0–98.8)	9.68 (2.53–26.9)	48.1 (34.5–62.0)	60.0 (17.0–92.7)	49.1
	2 of 3 findings	89.3 (70.6–97.2)	43.8 (26.8–62.1)	58.1 (42.2–72.6)	82.3 (55.8–95.3)	65.0
	3 of 3 findings	32.1 (16.6–5.24)	78.1 (60.0–90.1)	56.2 (30.6–79.2)	43.2 (28.7–58.9)	56.7

**Table 3:** Areas under the curve (AUC) and associated confidence intervals (CI) from receiver operating characteristic curves for SUV_max_, SUV_peak_, and tumor-to-liver ratio (TLR). Using these present data, cutoff points were selected to optimize sensitivity and specificity. Our cutoff point for SUV_max_ differed slightly from that established in the previous literature, so the sensitivity and specificity of the previous cutoff point were calculated using the present data.

	AUC	CI	Cutoff	Sensitivity	Specificity
SUV_max_	0.85	0.73–0.96	5.3	91.2	70.0
SUV_peak_	0.83	0.71–0.96	4.5	91.7	65.0
TLR	0.84	0.71–0.97	3.0	91.3	68.4

## Data Availability

The datasets generated and analyzed during the current study are not publicly available for privacy of the subjects but may be available from the corresponding author on reasonable request.
